# The Relationship Between Psychological Well-Being and Autonomy in Young People According to Age

**DOI:** 10.3389/fpsyg.2020.559976

**Published:** 2020-12-10

**Authors:** Ángel De-Juanas, Teresita Bernal Romero, Rosa Goig

**Affiliations:** ^1^Faculty of Education, Universidad Nacional de Educación a Distancia, Madrid, Spain; ^2^Faculty of Psychology, Universidad Santo Tomás, Bogotá, Colombia

**Keywords:** autonomy, psychological well-being, transition to adulthood, young people, positive psychology, self-organization

## Abstract

Psychological well-being manifests itself in all aspects of human activity and is essential to understanding whether young people experience life satisfaction and whether, as they mature, well-being can be associated with different levels of personal autonomy. This quantitative study was developed within the framework of international research on young people’s autonomy in the transition to adulthood. Its main objectives were to analyze the relationship between psychological well-being and autonomy and examine potential variations between the two variables according to age. To this end, Ryff’s Psychological Well-Being Scale and the Transition to Adulthood Autonomy Scale (EDATVA) designed by Bernal et al., were used with a sample of 1,148 young people aged 16–21 from Madrid, Spain, and Bogotá, Colombia. The results show that almost all the dimensions on the Psychological Well-Being Scale correlate significantly and positively with the dimensions on the EDATVA scale. Specifically, moderate correlations were obtained between *self-organization* on the EDATVA scale and *purpose in life* (*r* = 0.568; *p* = 0.01) and *environmental mastery* (*r* = 0.447; *p* = 0.01) on the Psychological Well-Being Scale. In turn, *autonomy* on Ryff’s scale obtained the highest correlation (*r* = 0.382; *p* = 0.01) with *understanding context* on the EDATVA scale. It was also found that the older 18–21 age group obtained higher scores than the younger 16–17 age group in all dimensions on both the EDATVA and the Psychological Well-Being Scale. Earlier studies endorse the results found in this research, especially the differences in the scores for both scales according to age groups. This opens avenues for future research to analyze the relationship between psychological well-being and autonomy as independent variables in other sectors of the population.

## Introduction

Advances in positive psychology have given rise to heightened interest in psychological well-being across various disciplines (Henn et al., 2016; Hides et al., 2016). This has led to the scientific literature taking an approach to the construct from two polarized perspectives. In the first one, psychological well-being is construed from a hedonic perspective, the result of an internal state that the individual experiences on a subjective temporal plane, associated with high levels of positive affect and life satisfaction (Weiss et al., 2016; Opree et al., 2018). Consequently, it focuses on subjective experiences of well-being specifically relating to happiness, life satisfaction, and positive affect (Henn et al., 2016). In contrast, in the second perspective, psychological well-being is construed from a eudemonic perspective as a process of self-realization through which individuals evolve over time. Subsequently, it is not associated with results but with capacities (Díaz et al., 2015; Berzonsky and Cieciuch, 2016; Disabato et al., 2016; Urquijo et al., 2016).

In line with the second perspective, Ryff (2014; 2018; 2019) designed a series of indicators based on the theory of positive human functioning that are consistent with a eudemonic perspective on happiness. To this end, she configured a composite and multidimensional model, the Psychological Well-Being Scale, that has been used as the basis for this study, comprising *self-acceptance*, *positive relations with others*, *autonomy*, *environmental mastery*, *personal growth*, and *purpose in life*. These dimensions focus on the different capacities of individuals to regulate their own behavior, assume the demands of the context, develop individual potential by maintaining *positive relations with others*, accept their own limitations while maintaining a positive attitude, and establish meaning and direction in their own lives (Keyes et al., 2002; Viejo et al., 2018; Gómez-López et al., 2019). In turn, these dimensions, and in particular *environmental mastery*, are closely related to the individual’s sense of autonomy and capacity for self-determination and independence (Rosa-Rodríguez et al., 2015). As a result, these indicators are often referred to as “health assets” given that they affect young people’s physical and mental health and, ultimately, the development of their behavior (Chen et al., 2019).

Moreover, it has been determined that sociodemographic correlates, such as age, are linked to psychological well-being in various ways. It has also been determined that psychological well-being is related to psychological constructs, such as life experiences, emotional intelligence, and personality traits, and that there is a significant positive correlation between level of education and psychological well-being—in reference to *personal growth* and *purpose in life* (Bucchianeri et al., 2016; Henn et al., 2016; Butler-Barnes et al., 2017). In turn, Mayordomo et al. (2016) found a positive correlation between age and level of psychological well-being, which might be the result of successful adaptation to the social environment. In this regard, these authors specify that adaptability can be defined as the flexibility to choose how to govern one’s own behavior. In contrast, the progressive loss of psychological well-being could denote exposure to threats and challenges which the individual in question cannot resolve due to lack of adequate skills (Bradshaw et al., 2013).

In the transition to adulthood, psychological well-being evolves to the extent to which the individual is capable of successfully interacting with their environment and assuming the vital challenges inherent to the different stages in life (Vera-Villarroel et al., 2013; Bluth et al., 2017; Gómez-López et al., 2019). To this end, García-Moya et al. (2015) suggest that psychological well-being can be promoted through the generation of positive experiences in young people’s environments which help them perceive their purpose and direction in life and set their own goals. However, promoting psychological well-being requires identifying which variables interfere with or condition well-being.

In this context, autonomy is seen as one of the dimensions that constitute psychological well-being (Ryff, 1989). Consequently, the interaction between both variables is often taken for granted, as autonomy is considered an integral construct of well-being that describes people’s positive functioning based on their ability to maintain their individuality in different contexts and situations. As a result, the study of autonomy has been approached from various disciplines, including psychoanalysis, philosophy, pedagogy, politics, psychology, and biology, *inter alia*. Importantly, all agree that autonomy is a complex concept in which different perspectives can be identified and grouped. One such group is the one that focuses on the study of an individual’s ability to make decisions or govern their actions according to their own criteria, which are independent from external influences (Garberoglio et al., 2017). In a broad sense, this perspective emphasizes the development and construction of the criteria used by individuals to make decisions and act in consequence. Other perspectives on autonomy recognize the influence of different scenarios in which individuals construct decision-making processes. Similarly, some authors defend that within decision-making and the very construct of autonomy, the idea of interdependence between individuals takes on a leading role (Álvarez, 2015; Seidl-De-Moura et al., 2017).

Personal autonomy as an integral part of quality of life has been studied as a process that develops throughout an individual’s lifetime. Thus, several studies in this respect show that the older a person is, the greater the degree of autonomy (Barbosa and Wagner, 2015). In this regard, Campione-Barr et al. (2015) analyzed the effect of age on young people’s autonomy and the impact of siblings’ ordinal positions within the family. The authors conclude that both age and the organization of fraternal subsystems are important in the development of autonomy in individuals. In the same vein, Barbosa and Wagner (2015) found that higher levels of autonomy are found in groups of older young people. In this regard, it was determined that the desire for autonomy increases during adolescence regardless of gender (Alonso-Stuyck and Aliaga, 2017). However, Mayordomo et al. (2016) conducted a study with more than 700 participants distributed in three different age groups—young people, adults, and older adults—which revealed that there were no significant differences in autonomy between adults and older adults on Ryff’s Psychological Well-Being Scale, although both groups scored higher than the group of young people.

These studies highlight the importance of research on young people’s autonomy which could lead to a better understanding of their life cycle development processes, as well as the ways in which they assume responsibility in life and for their own well-being (Davies et al., 2015; Li and Hein, 2019). Taking into account the aforementioned literature, our study is based on the approach designed by Bernal Romero et al. (2020), in which autonomy is considered as a wide-ranging, complex construct that involves the capacity to ask oneself questions, reflect on one’s life in relation to others, make interdependent decisions and assume the consequences, and organize oneself in relation to others and society. In consequence, Bernal Romero et al. (2019) designed a model, called the Transition to Adulthood Autonomy Scale (EDATVA), comprising four fundamental dimensions for understanding autonomy in young people: *self-organization*, *understanding context*, *critical thinking*, and *sociopolitical engagement*. This approach has been incorporated to this study with the aim of determining the potential relationships between young people’s psychological well-being and autonomy in their transition to adulthood.

## Materials and Methods

### Specific Objectives

This article presents selected partial results from research performed in Spain and Colombia as part of a wider study on the autonomy of young people and psychological well-being. The main objective of the study was to analyze the relationships between young people’s psychological well-being and autonomy. This responds to the hypothesis (H1) that there are statistically significant relationships between psychological well-being and autonomy for the sample participating in the study. The second objective was to examine the differences between psychological well-being and autonomy according to age by establishing two groups: young people under 18 and those 18 and over. This responds to the hypothesis (H2) that there are statistically significant differences in both the dimensions of psychological well-being and autonomy as a function of age, the assumption being that participants in the older age group will have higher scores.

For practical reasons and according to the nature of this descriptive study, a quantitative methodology and an *ex post facto* pre-experimental design were used.

### Participants

The field work was performed from late 2018 to early 2019. An incidental non-probabilistic sampling was performed in which 1,148 young people aged 16–21 were selected (*M* = 18.20; SD = 1.80). Of the total, 60.3% were female and 39.7% were male. The percentage of adolescents aged 16–17 was 39.7%, while those aged 18–21 at the time of the study represented 60.3%. The sample was divided into these two subgroups, given that the legal age is 18 in both countries. Most of the young people were Colombian (55.7%, from Bogotá), while the rest were Spanish (44.3%, from Madrid).

Most of the participants were studying in high schools and universities. Data were also collected from young people who were employed, as well as from participants who were under the tutelage of child protection services. As an exclusion criterion, it was decided not to include those individuals who had functional, physical, or mental difficulties that prevented them from participating in the study.

### Tools Used

Two methods were used to perform the study. The first one, Ryff’s Psychological Well-Being Scale adapted in Spanish by Díaz et al. (2006), is a multidimensional scale that assesses the factors that contribute to an individual’s psychological well-being. It has 39 items with responses from 1 (strongly disagree) to 6 (strongly agree) on a Likert-type assessment scale, with six dimensions corresponding to the positive attributes of psychological well-being established by Ryff (1989). The first dimension is *self-acceptance* or fostering a positive attitude toward one’s self. This dimension presents six items (α = 0.83) and measures self-esteem and the awareness of one’s own strengths and weaknesses. The second is *positive relations with others*. This dimension also has six items (α = 0.81) and measures an individual’s ability to maintain trusting, stable, and intimate relationships. The third is *autonomy*, which has eight items (α = 0.73) that measure an individual’s capacity to maintain their individuality in different contexts and situations with determination, independence, and personal authority. The fourth is *environmental mastery* and has six items (α = 0.71); it explores whether individuals consider themselves to be efficient at managing and controlling their daily responsibilities. This dimension is intimately related to the locus of control, self-efficacy, and the capacity to generate favorable environments that enable the individual to satisfy their needs and desires. The fifth dimension is *personal growth*, which has seven items (α = 0.68) and examines an individual’s capacity to evolve, develop their potential, and continue to grow on the basis of positive learning. Finally, the sixth dimension is *purpose in life* which comprises six items (α = 0.83) and measures an individual’s positive psychological well-being by analyzing their capacity to set goals, establish objectives, maintain the level of motivation to achieve them, and give purpose to their life.

The second method, which was used to measure young people’s autonomy, is the Transition to Adulthood Autonomy Scale (hereinafter EDATVA) designed by Bernal Romero et al. (2020). It has a total of 19 items composed of statements with responses on a Likert-type scale with four options (1 = strongly disagree and 4 = strongly agree). The items are grouped in four dimensions. The first dimension is *self-organization*, which comprises six items (α = 0.80) that examine whether young people successfully plan their time and the processes in which they participate. This capacity requires young people to make personal choices according to their priorities (Lammers et al., 2016; Bernal Romero et al., 2019). The second dimension is *understanding context*, which has four items (α = 0.74) and explores young people’s interaction with their environment, which leads to them becoming more autonomous (Reis et al., 2018). The third dimension is *critical thinking*, which has five items (α = 0.70) and aims to measure an individual’s competence in establishing their position and guaranteeing their interests in relation to different social situations that affect them and/or may interest them (Van Petegem et al., 2015). Finally, the fourth dimension is *sociopolitical engagement*, which has four items (α = 0.77). This dimension measures young people’s commitment to the society they belong to, the processes of community participation, and the political rights of contemporary citizens (Young, 2017). As a whole, the model obtained a Cronbach’s alpha of 0.84.

### Procedure and Data Analysis

This study adheres to the Declaration of Helsinki (64th WMA, Brazil, October 2013) and was approved by the Human Research Ethics Committee of the universities involved in the research. The application of the models was systematic, and data were collected using a pencil and paper format mostly during school hours. Approval and informed consent were obtained from the participating centers, as well as the legal guardians and the participants themselves. Once the data had been collected, the responses were coded, arranged, and recorded in a computer database for subsequent statistical processing.

Descriptive statistics of the participants’ general characteristics were then calculated. Pearson’s correlation coefficient was also calculated for the study’s first objective, aimed at determining the relationship between the dimensions of the well-being scale and EDATVA for the sample as a whole. For the second objective, the assumptions of the statistical tests were verified using common procedures (e.g., Kolmogorov–Smirnov test, Shapiro–Wilk tests, Levene’s test, histograms, and Q-Q and P-P diagrams for normality). Mean difference analyses were performed for the two groups to determine potential differences according to age. The effect sizes were estimated using Cohen’s *d*. Non-parametric tests were used in those cases where assumptions of normality were not met, specifically the Mann–Whitney *U* test with the Bonferroni correction.

All statistical analyses were performed using the SPSS version 25.0 statistical package for Macintosh (IBM^®^, SPSS^®^, Statistics 25). The statistical significance level was set at <0.05.

## Results

The following are the results for the first objective, in which the relationships between the dimensions of the Psychological Well-Being Scale and EDATVA were analyzed. Table 1 shows the results of Pearson’s correlation coefficient for the different dimensions of the Psychological Well-Being Scale and EDATVA. Significant correlations with positive directionality were found between almost all dimensions on both scales. High correlations were obtained in *self-organization* on the EDATVA scale and *purpose in life* (*r* = 0.568; *p* = 0.01) and *environmental mastery* (*r* = 0.447; *p* = 0.01) on the Psychological Well-Being Scale. These results give rise to moderate correlations and show that the higher young people score in *self-organization*, the higher they score in *purpose in life* and *environmental mastery*.

**TABLE 1:** Correlation between autonomy and psychological wellbeing.

	**Self-organization**	**Understanding context**	**Critical thinking**	**Socio-political engagement**	**Total EDATVA**
Self-acceptance	0.367**	0.275**	0.167**	0.145**	0.325**
Positive relations with others	0.066*	0.215**	0.063*	0.091**	0.151**
Autonomy	0.199**	0.382**	0.200**	0.038	0.274**
Environmental mastery	0.447**	0.333**	0.251**	0.160**	0.406**
Personal growth	0.354**	0.356**	0.279**	0.097**	0.369**
Purpose in life	0.568**	0.340**	0.276**	0.186**	0.466**
Total wellbeing	0.437**	0.426**	0.275**	0.159**	0.441**

*ρ < 0.05. **ρ *< 0.01*.

The dimension *understanding context* obtained the highest correlations with *autonomy* (*r* = 382; *p* = 0.01) and *personal growth* (*r* = 0.356; *p* = 0.01) on the Psychological Well-Being Scale.

*Critical thinking* obtained the highest correlations with *personal growth* (*r* = 0.279; *p* = 0.01) and *purpose in life* (*r* = 0.276; *p* = 0.01).

*Sociopolitical engagement* obtained the lowest overall correlations, while *purpose in life* obtained the highest (*r* = 0.186; *p* = 0.01).

The overall results show that all the dimensions on the EDATVA scale correlate significantly with the dimensions on the Psychological Well-Being Scale. In this regard, *self-organization* obtained the highest total correlation (*r* = 0.437; *p* = 0.01), followed by *understanding context* (*r* = 0.426; *p* = 0.01). In turn, *purpose in life* obtained the highest correlation between the Psychological Well-Being Scale and EDATVA (*r* = 0.466; *p* = 0.01), followed by *environmental mastery* (*r* = 0.406; *p* = 0.01). Lastly, the total for both scales gave a result of 0.441 (*p* = 0.01).

### Comparison of Mean Values Between the Psychological Well-Being Scale and EDATVA According to Age

In order to determine the potential differences for each of the scales according to age, tests were performed to contrast central tendency scores.

The Psychological Well-Being Scale is represented in Table 2, which shows the size of each group, mean values, and standard deviation. For both groups of young people, the highest average scores were found in *autonomy* and *personal growth*. Moreover, in all cases, the results show that those aged 18–21 obtained higher scores than those aged 16–17. However, the results of Student’s *t*-test show statistically significant differences in five of the six dimensions of psychological well-being and for the scale’s total score. No statistically significant differences were found in *positive relations with others*.

**TABLE 2:** Differences on the psychological well-being scale according to age.

	**Age**							
	**Under 18 (*n* = 450)**		**18–21 (*n* = 679)**					
					
	***M***	**SD**	***M***	**SD**	***df***	***t***	***p***	***d***
Self-acceptance	24.37	5.99	25.65	5.49	1,127	–3.70	0.000	0.223
Positive relations with others	25.16	6.45	25.70	5.92	1,127	–1.42	0.157	−
Autonomy	33.52	7.16	34.57	6.57	1,127	–2.53	0.011	0.153
Environmental mastery	24.16	5.12	26.08	5.01	1,127	–6.24	0.000	0.380
Personal growth	31.59	5.30	33.42	5.12	1,127	–5.77	0.000	0.351
Purpose in life	25.97	6.05	27.81	5.37	1,127	–5.66	0.000	0.322
Total psychological well-being	164.39	27.66	173.23	24.46	1,127	–5.64	0.000	0.339

In relation to EDATVA, Table 3 shows the size of each group, the mean values, the Mann–Whitney *U* statistic, the statistical classification and significance, and the effect size. In turn, the results show that the 18–21 group obtained the highest average scores in *self-organization* and *critical thinking*. For the under 18 group, the dimensions with the highest average scores were *sociopolitical engagement* and *understanding context*. Similarly, the effect of age on *autonomy* is also shown. As can be seen, the older group of young people obtained higher average scores. These differences are statistically significant in all dimensions except *sociopolitical engagement*.

**TABLE 3:** Differences in perceived autonomy according to age.

	**Age**					
	**Under 18 (*n* = 450)**	**18–21 (*n* = 679)**				
	**Mean value**	**Mean value**	***Z***	***U***	***p***	***D***
Self-organization	521.14	604.07	–4.180	133,379	0.000	0.368
Understanding context	546.98	586.93	–2.026	145,136.50	0.043	0.144
Critical thinking	516.07	607.43	–4.608	131,074	0.000	0.312
Sociopolitical engagement	550.18	584.81	–1.747	146,592	0.081	0.113
Total EDATVA	519.10	605.42	–4.335	132,450	0.000	0.300

## Discussion

The results of our study confirmed the hypothesis (H1) that there are statistically significant relationships between the dimensions on the Psychological Well-Being Scale and the autonomy dimensions on the EDATVA scale. A significant positive correlation was identified between the total of the Ryff’s scale and the total of the EDATVA scale. We consider the relationship between the two scales to be highly significant, given that autonomy is only considered as one of the factors of psychological well-being and conceived as a multidimensional construct in other studies (Panahi et al., 2013; Roslan et al., 2017; Gao and McLellan, 2018; Ryff, 2019). In our study, psychological well-being and autonomy are considered as two different constructs. In the Psychological Well-Being Scale, autonomy is construed as an individual’s capacity for self-regulation independent of others, whereas in EDATVA, it is conceived as a construct defined as a complex process of reflection and decision-making interdependent of others, constituting a relational construct (Bernal Romero et al., 2020). This conceptual difference highlights the importance of establishing relationships between the two constructs, as in this study.

Earlier studies had already documented correlations between psychological well-being and autonomy, conceiving both concepts as independent processes. Thus, studies by Rivas et al. (2012) and Romero et al. (2013) correlated psychological well-being with perceived autonomy, taking into account two dimensions of the latter: choice and volitional intention. Both studies found that the greater the perceived autonomy, the greater the level of well-being, with the exception of the volitional dimension of autonomy. In turn, other studies also coincide with our study by determining that increased levels of autonomy are associated with higher levels of well-being (Ratelle et al., 2013; Weiting, 2014; De Leersnyder and Kim, 2015).

In this study, we also found significant positive relationships between almost all the dimensions on the two scales. The correlations are higher between *self-organization* on the EDATVA scale and *purpose in life* and *environmental mastery* on the Psychological Well-Being Scale. This suggests that those individuals whose life goals and objectives are clearer and who are better able to control their environment according to their needs may also be better at organizing themselves to make better decisions, which gives them more autonomy (Valle et al., 2011).

Interestingly, *autonomy* on the Psychological Wellness Scale presents three positive and significant correlations with the EDATVA dimensions: the highest correlations were obtained with *understanding context*. In this regard, the results suggest that the more younger people are concerned about their development and giving direction to their lives, the more they are able to defend their ideas and uphold their decisions. These findings are similar to those of other studies (Morales and González, 2014; Rodríguez-Fernández et al., 2016; Valle et al., 2019).

Other results to be considered are that the lowest correlations in our study were found between all the dimensions on the Ryff’s scale and *sociopolitical engagement* on the EDATVA scale. This can be attributed to the fact that the Psychological Well-Being Scale focuses on intrasubjective aspects, while the EDATVA focuses on intersubjective aspects. Specifically, *sociopolitical engagement* involves a tendency to construct autonomy in relation to others, rather than to oneself. Thus, Arnett (2014) describes how young people are more focused on their processes of individuation, leaving aside the effects of their decisions on the context. In contrast, Valle et al. (2019) found that psychological well-being is related to the relationships established by individuals in public domains. Based on the difference in the results, future research needs to study this aspect further (García-Alandete et al., 2018).

Our study’s second objective, namely the hypothesis (H2) of the existence of statistically significant differences both in the dimensions of psychological well-being and in the dimensions of autonomy according to age, was also confirmed. The results prove the existence of variations in psychological well-being according to age coinciding with other studies (Ryff, 1989, 1991, 2014, 2019; Ryff and Keyes, 1995; Springer et al., 2011). Specifically, we found that the older group scored higher than the younger group. In this regard, Bluth et al. (2017) state that during adolescence, well-being tends to decrease due to the changes experienced during that particular period, which could partly explain our findings. However, in relation to the positive correlations with other dimensions on the Psychological Well-Being Scale, no differences were found between the two age groups. These results are consistent with the studies by Roecke et al. (2009) and Carstensen et al. (2011), who determined that affective relationships are more stable the older the individual.

On the other hand, the differences caused in autonomy as an effect of age during transition to adult life must also be taken into account. In this respect, our findings indicate that there is a significant increase in the levels of autonomy in older individuals. These results coincide with the ones obtained by Ryff (1989, 2014), Barbosa and Wagner (2015); Campione-Barr et al. (2015), Mayordomo et al. (2016), and Alonso-Stuyck and Aliaga (2017). In line with the said studies, our findings show that young people over the age of 18 achieved a higher average score and that their highest average ranking was in *self-organization* and *critical thinking.* Among other factors, this result can be explained by the fact that during this period young people are making very important decisions that require looking toward the future, for example in choosing what they are going to study at university (Kiang and Bhattacharjee, 2018).

It should also be noted that the results for *sociopolitical engagement* on the EDATVA scale were not significant. According to Ryff (1989) and Barrera et al. (2019), autonomy involves adopting personal standards that allow the individual to take control of their decisions and discard external influences in relation to personal choices. However, in the case of EDATVA, these influences are taken into account, especially in *sociopolitical engagement*. Therefore, it is noteworthy that the differences according to age are not maintained in this dimension, which involves the individual reflecting on the consequences of their decisions on others. From a developmental perspective, it might be expected that this level of reflection would increase with age as in the other dimensions of autonomy, but this was not the case with the sample in this study. Likewise, Parés and Subirats (2016) research findings corroborate that young people’s political behavior is diverse, and the differences are not the result of age. Consequently, we believe that this is an area that requires future research.

Lastly, although the data in our study confirmed our hypotheses, we should not ignore its limitations. This study dealt with two particularly complex objectives within the concept of young people’s transition to adulthood. Future research should take into account other sectors of the population when exploring the relationship between psychological well-being and autonomy: different age ranges, problems, nationalities, and contexts.

## Data Availability Statement

The raw data supporting the conclusions of this article will be made available by the authors, without undue reservation.

## Ethics Statement

The studies involving human participants were reviewed and approved by the UNED Ethical Committee; USTA Ethical Committee. Written informed consent to participate in this study was provided by the participants’ legal guardian/next of kin.

## Author Contributions

ÁD-J coordinated the project, designed the database, completed the statistical analysis, and reviewed the final version of the article. TB and RG prepared the introduction and theoretical framework, and wrote the discussion section. RG reviewed the references section. All authors wrote the initial version of the article.

## Conflict of Interest

The authors declare that the research was conducted in the absence of any commercial or financial relationships that could be construed as a potential conflict of interest.
